# Supplementary material for: Local Selection Shaped the Diversity of European Maize Landraces
Source: Mol Ecol. 2025 Mar 17;34(24):e17720. doi: 10.1111/mec.17720 (PMC12717992; doi:10.1111/mec.17720)
Supplement: Supplementary file 1 — Figure S1. [file MEC-34-e17720-s001.pdf]

Supplement

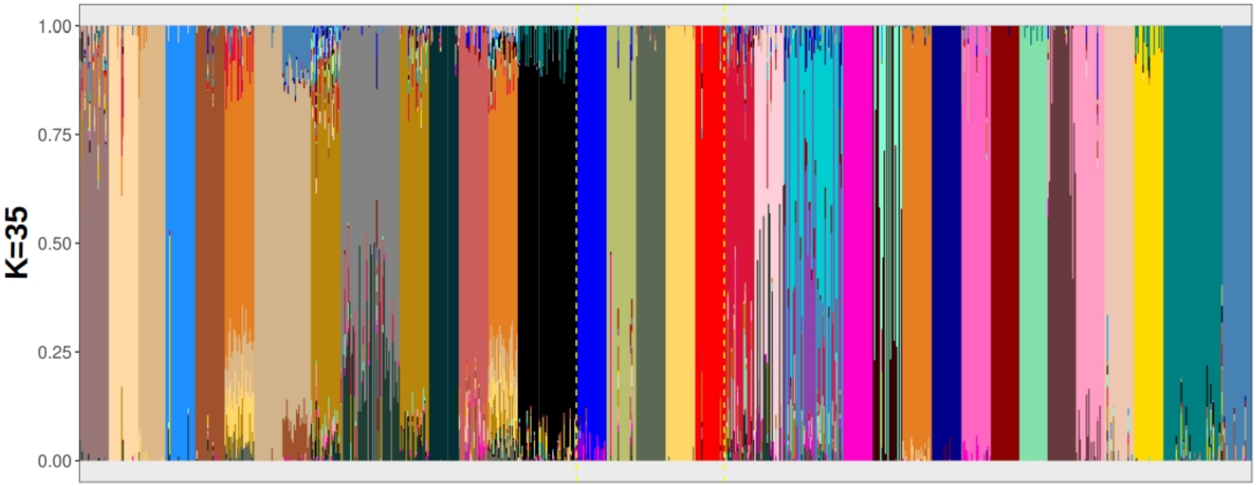

Figure S1: **Structure analysis of the European maize landraces.** Result for K=35, which was the optimal number of clusters are shown. The populations are ordered as in the F_{ST} heatmap (Figure 2). The dashed yellow lines in both plots indicate where each F_{ST} group starts and ends.

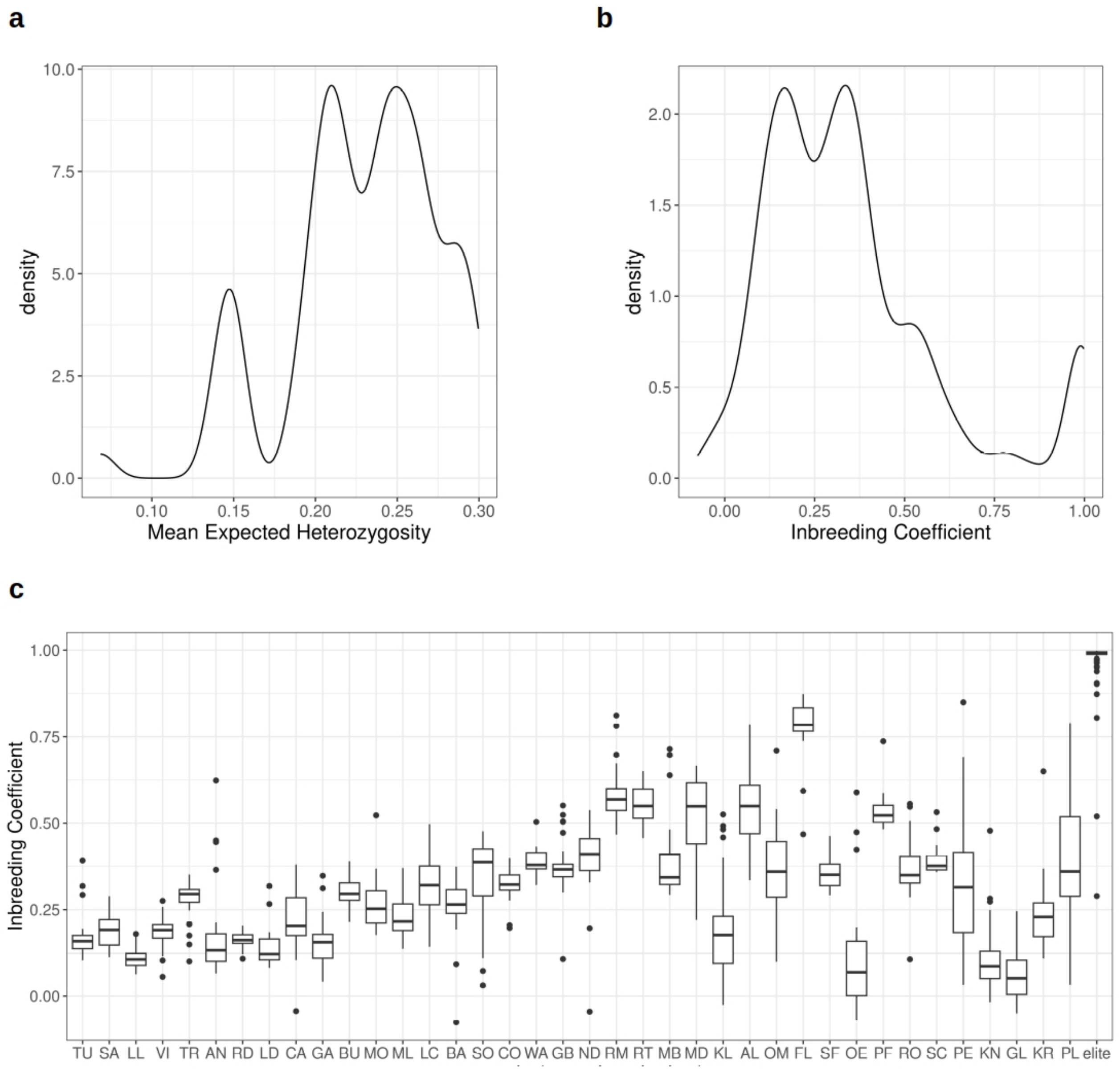

Figure S2: **Genetic diversity within European maize.** a. The density of the mean H_{Exp} for all populations is shown. b. The density of the F_{IS} values for each individual of all landraces and elite lines. c. The F_{IS} values per population, ordered from West to East with the elites depicted at the far right of the plot.

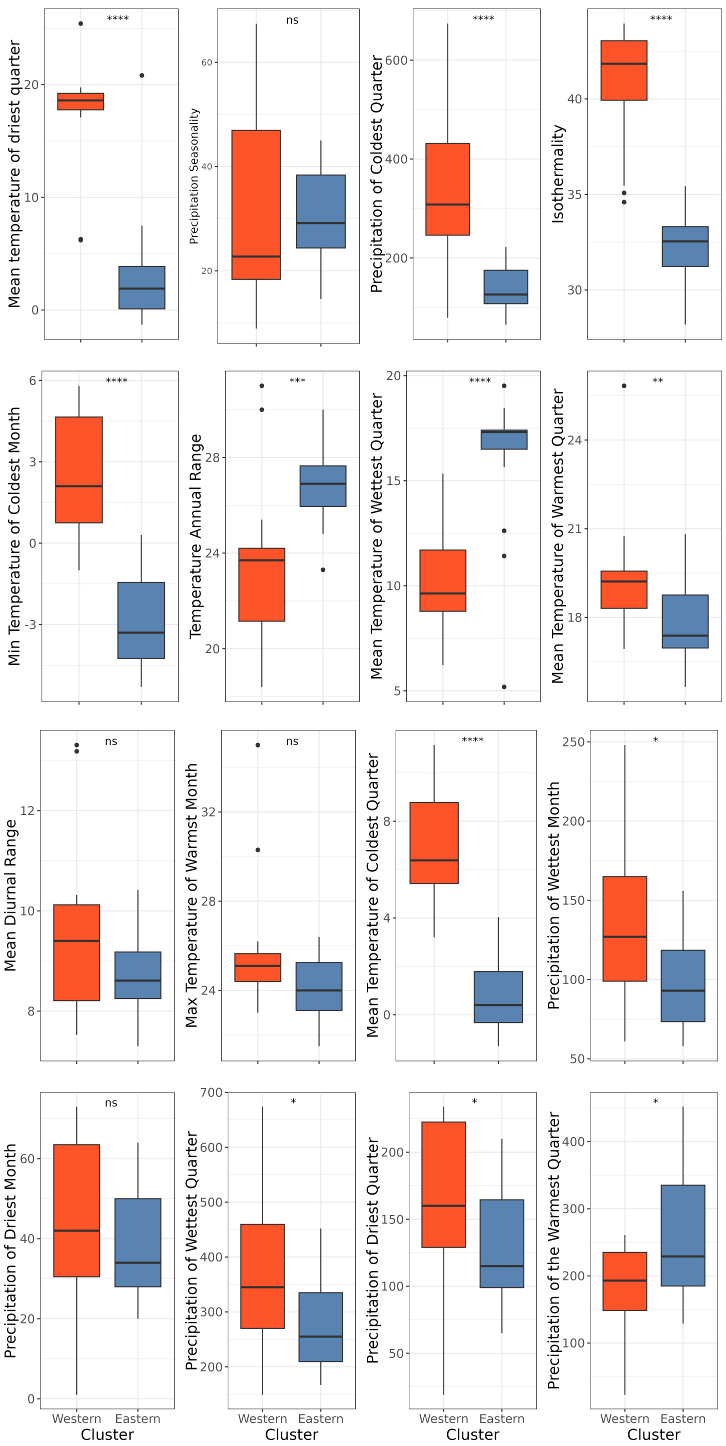

Figure S3: **Environmental diversity between the Eastern and Western European population clusters, comparing the historical bioclimatic factors bio1-bio19. The significance level for each parameter is indicated with 'ns' for p ¿ 0.05, * for p ¡=0.05, ** for p¡=0.01, *** for p ¡=0.001 and **** for p¡=0.0001.**

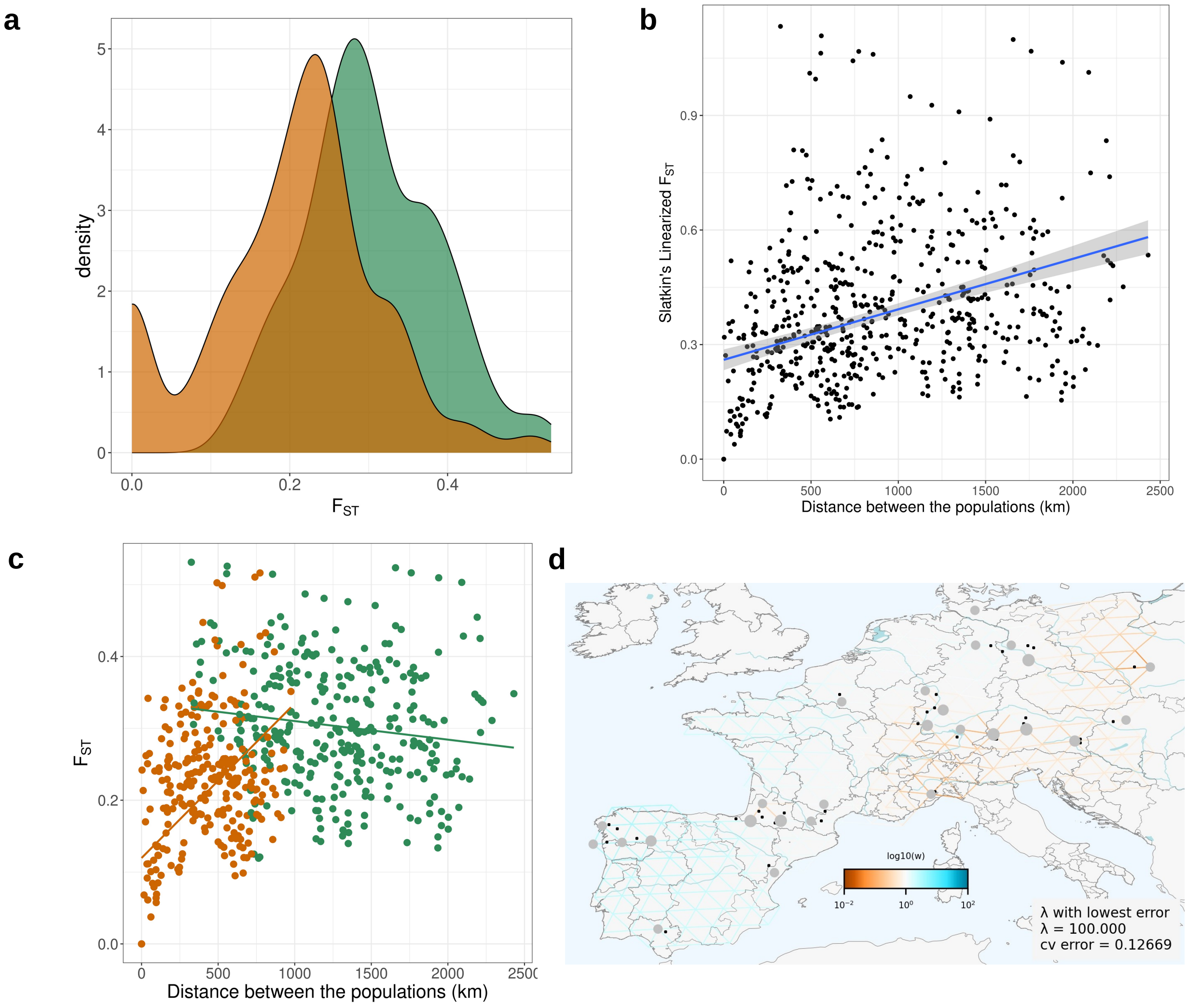

Figure S4: **a. The distributions of pairwise F_{ST} values for all the pairs within the same cluster (orange), or between the two clusters (green). b. Slatkin's Linearized F_{ST} against the distance between the populations in kilometers. c. F_{ST} values are plotted against the environmental distance between the populations. The values in green show pairs of populations belonging to different clusters and orange pairs of populations belonging to different clusters. d. Effective migration rate within Europe. The grid is colored blue when estimated effective migration rate lower than expected and red when it is higher than expected.**

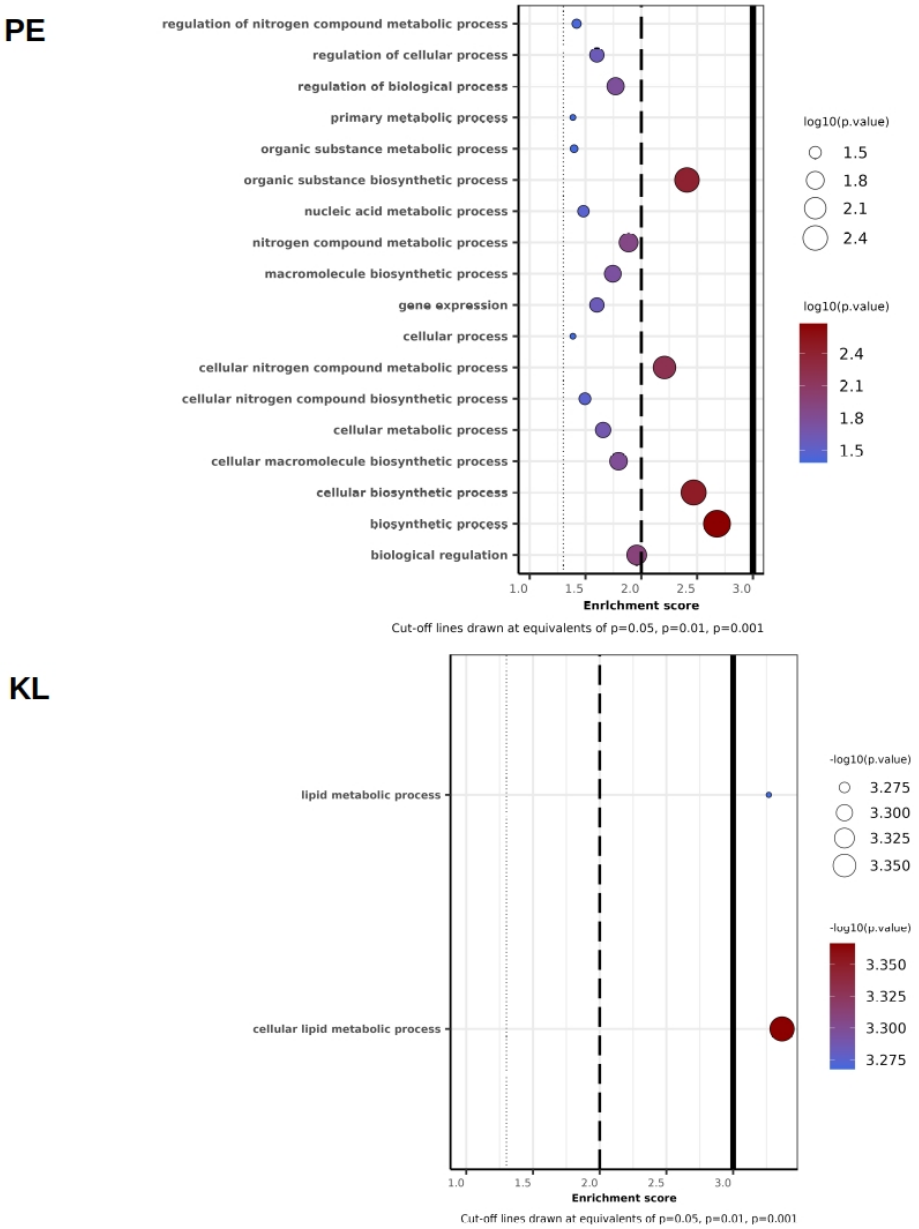

Figure S5: **Enriched Gene Ontology categories for the genes closest to the markers with selection signal for Petkuser Ferdinard Rot DH lines (PE) on the top and Kemater Landmais Gelb DH lines (KL) on the bottom.**

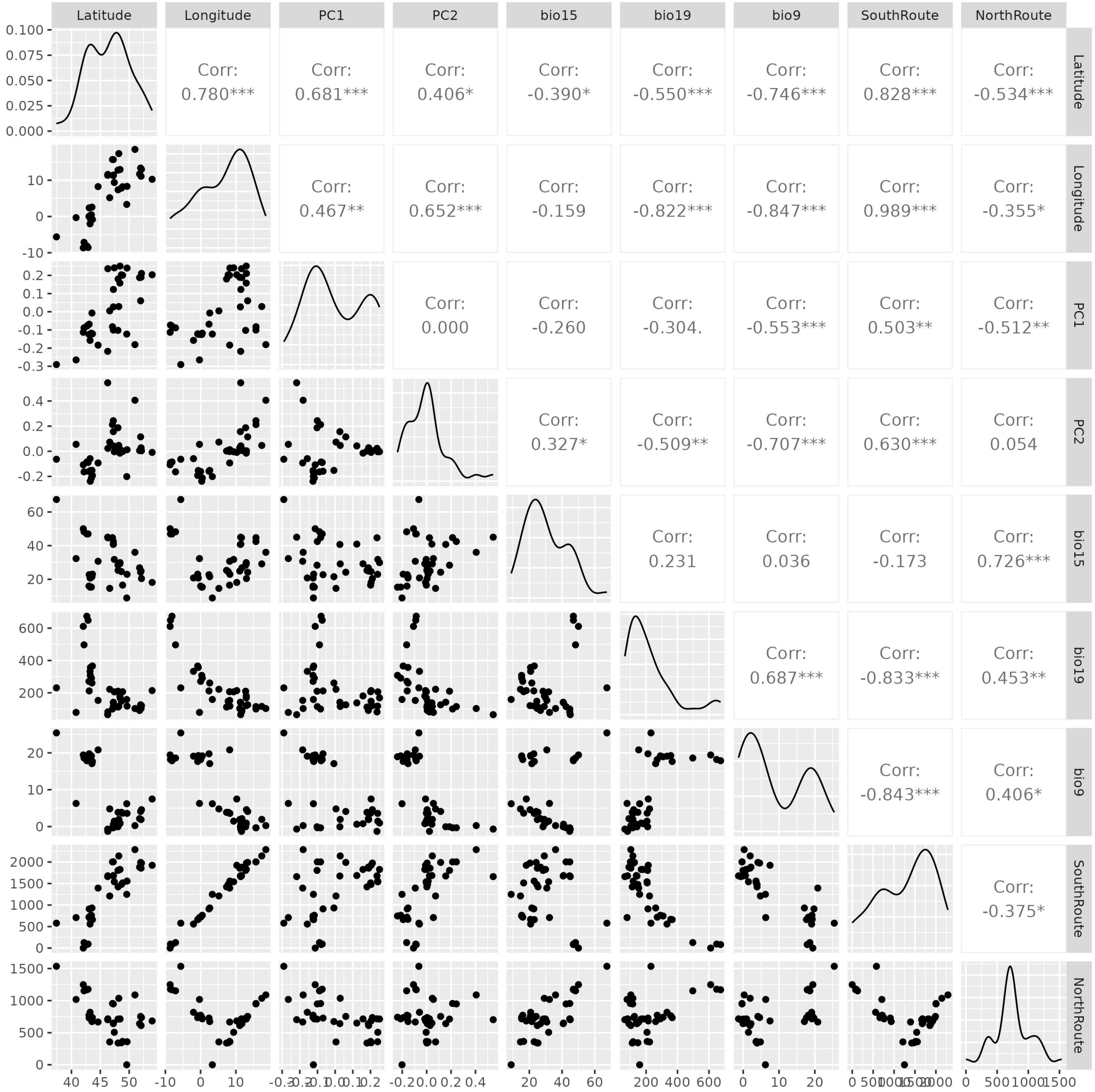

Figure S6: **Environmental and Genetic diversity parameters explaining genetic variation based on a redundancy analysis.**

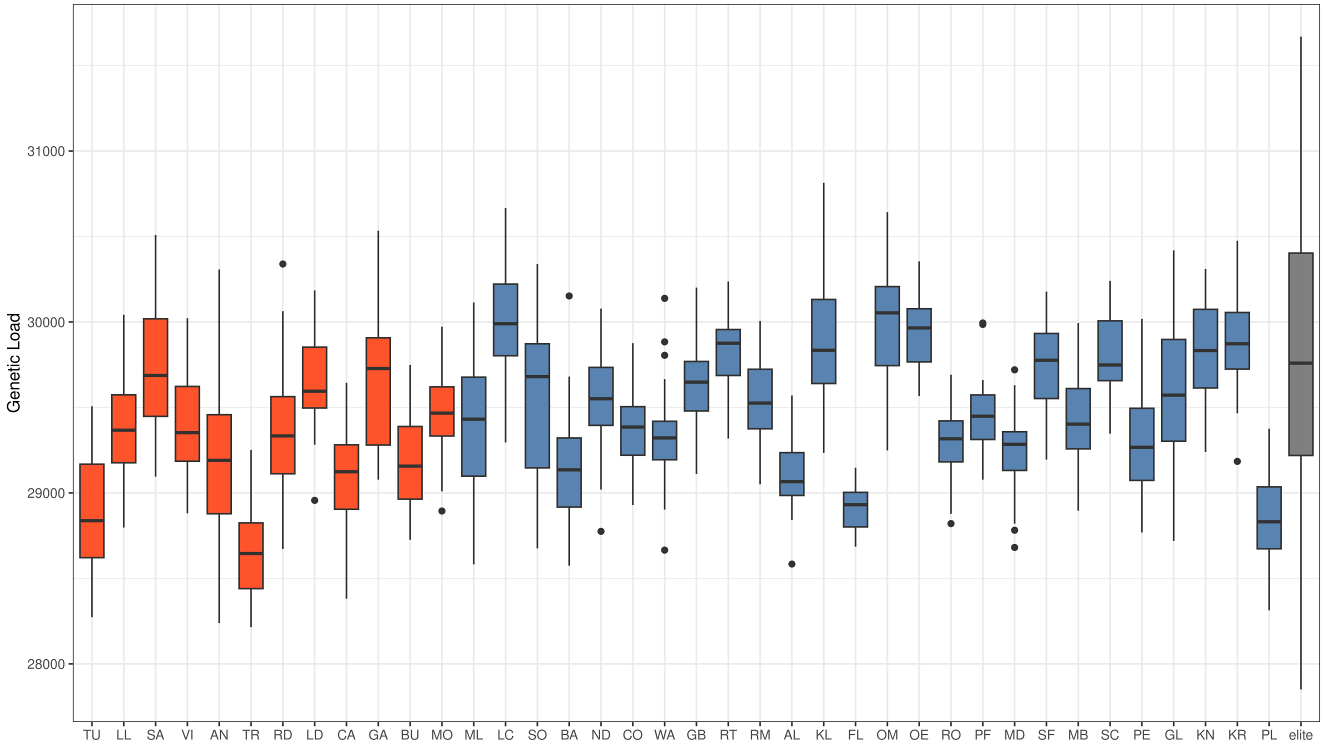

Figure S7: **Genetic load per European landrace population ordered by their distance from the hypothetical Southern introduction point. In red, all populations belonging in the Western cluster, in blue populations of the Eastern cluster and in grey the elite lines.**
